# Decomposing Working Memory in Recurrent Major Depression: Impaired Encoding and Limited Maintenance Immune-to-Encoding Constraint

**DOI:** 10.3390/brainsci13010038

**Published:** 2022-12-24

**Authors:** Zhitang Chen, Zheng Dou, Hui Xu, Zhenghua Wang, Suhua Zeng, Xiangyu Yang, Eiki Takahashi, Milos R. Popovic, Lihui Wang, Weidong Li

**Affiliations:** 1 Key Laboratory for the Genetics of Development and Neuropsychiatric Disorders (Ministry of Education), Bio-X Institutes, School of Life Sciences and Biotechnology, Shanghai Jiao Tong University, Shanghai 200240, China; 2 Institute of Psychology and Behavioral Science, Shanghai Jiao Tong University, Shanghai 200000, China; 3 WLA Laboratories, World Laureates Association, Shanghai 200233, China; 4 The Fourth People’s Hospital of Wuhu, Wuhu 241002, China; 5 Toronto Rehabilitation Institute and The KITE Research Institute, University Health Network, University of Toronto, Toronto, ON M5S 2E8, Canada; 6 Shanghai Key Laboratory of Psychotic Disorders, Shanghai Mental Health Center, Shanghai Jiao Tong University School of Medicine, Shanghai 200000, China; 7 Shanghai Center for Brain Science and Brain-Inspired Intelligence Technology, Shanghai 200120, China

**Keywords:** recurrent major depression, working memory, encoding, maintenance, load

## Abstract

It is generally believed that working memory (WM) is dysfunctional in depression. However, whether this impaired performance originates from impaired encoding, maintenance or both stages is still unclear. Here, we aimed to decompose the abnormal characteristics of encoding and maintenance in patients with recurrent major depressive disorder (MDD). Thirty patients and thirty-nine healthy controls completed a spatial working memory task where the encoding time and the retention time could vary under different load levels. Encoding performance was assessed by comparing accuracies between short and long encoding times, and maintenance performance was assessed by comparing accuracies between short and long retention times. The results show a lower performance in depression than the controls. However, while the decreased accuracy by long retention (vs. short retention) was increased by a short encoding time in the control group, the retention performance of the depression group did not further suffer from the short encoding time. The generally impaired encoding, together with limited maintenance of immunity against the constrained encoding time, suggests a common bias for fixed internal processing over external processing in recurrent MDD. The paradigm provided in this study can be a convenient and efficient clinical test for assessing the WM encoding and maintenance function.

## 1. Introduction

Depression is one of the most prevalent psychiatric disorders, affecting more than 264 million people worldwide [1]. Despite the progress in clinical treatment, the risk of relapse is still high, with an estimated recurrence rate of higher than 50% [2]. Cognitive models of depression posited that the development and recurrence of depression are associated with biased cognitive processing of both external (e.g., a negative event) and internal (e.g., a negative belief) information [3,4,5]. Importantly, depressive populations not only show cognitive bias for negative information, but also show general deficits in a broad range of cognitive functions, such as attention and executive control, even when no emotional information is concerned [6,7]. The cognitive deficits may act to potentiate the dysfunction of emotion regulation in depression [4].

Working memory (WM) is a core cognitive function that supports goal-directed behavior by providing an interface between perception, long-term memory and action [3,8]. WM is generally considered as comprising the encoding, temporal maintenance and manipulation of mental representation. E. J. Rose et al. used n-back tasks with different levels of task difficulty to explore the working memory performance of patients with depression. The results show that patients with depression had slower reaction times and decreased accuracy, while a faster response to tasks with higher levels of difficulty only occurred in healthy controls [9]. Using two verbal subtests and a performance subtest of the WAIS-R scale, Fouladi et al. [10] examined attention, working, and verbal memory in depressive patients., and found that the healthy group had better performance. Similarly, Stevan Nikolin et al. conducted a systematic review and meta-analysis and found that the accuracy of the n-back task of depressed patients was significantly reduced compared with the control group. Meanwhile, they also found that the clinical situation might aggravate depression-related working memory deficits [11]. Although previous studies have shown an impaired WM performance in depression [9,12], it remains unclear whether the WM deficits originate from impaired encoding, maintenance or both. 

To address these issues, in this study, we concurrently manipulated the encoding and retention difficulties in a spatial WM task and investigated how the difficulty in these two phases affects the following performance in recurrent major depressive disorder (MDD). Specifically, in this task, participants were first asked to memorize the locations of different shapes. After a retention interval without any sensory input, they were asked to report the location of one of the memorized shapes. The duration of the stimulus presentation and the retention interval could both be short or long, resulting in varying levels of difficulty for both encoding (short vs. long encoding) and retention (short vs. long retention). Regarding these within-group manipulations, the potential interaction between groups (depression vs. healthy controls) and the encoding/retention difficulty is not simply caused by the different motivation levels of the two groups to complete the task. We expected that short encoding and long retention would impair WM performance, resulting in lower accuracies in these two conditions than long encoding and short retention, respectively. In relation to our research question, depression-related encoding deficits predict that short encoding time should impair performance more in the depression group than in the control group. Similarly, maintenance deficits in depression predict that long retention impairs performance more in the depression group than in the control group. Given that the cognitive deficits in depression become evident as memory load increases [12], memory load was also manipulated in the present study by varying the number of the items that had to be memorized.

## 2. Materials and Methods

### 2.1. Participants

The sample size was determined based on a pilot study (Supplementary Materials, [13]) the availability of participants, and the inclusion and exclusion criteria. Patients were recruited from the outpatient psychiatric clinics of the Fourth People’s Hospital of Wuhu, China. Diagnosis was performed by licensed psychiatrists using structured interviews based on DSM-V [14]. Inclusion criteria for patients were: (1) aged 18–60 years old, right-handed, completed middle-school education (i.e., at least 9 years’ formal education); (2) diagnosed at least twice with MDD and experiencing a current episode; and 3) at least two months between the current episode and the previous episode. Patients were excluded if they met the criteria of schizophrenia, schizoaffective disorder, bipolar disorder, or anxiety disorder as a primary diagnosis. Thirty adult patients who met the above criteria participated in the experiment. The control group was recruited from the hospital and the community about the hospital through advertisements. Thirty-nine healthy participants met the following criteria were included in the control group: (1) aged 18–60 years old, right-handed, completed middle-school education; (2) did not meet the diagnostic criteria for MDD according to the clinical diagnosis; (3) reported no history of mental illness or neurological disease. The control group consisted of the accompanying people with patients, non-medical staff in the hospital, and those who live near the hospital. In addition to the clinical interview, both groups filled out the Beck Depression Inventory (BDI, [15]) prior to the experiment. The demographic and clinical characteristics of the two groups are shown in Table 1.

Informed consent was obtained from all participants prior to the experiment. We complied with APA ethical standards as well as the Declaration of Helsinki in the treatment of our participants. This study was approved by the Institutional Review Board for Human Research Protections of Shanghai Jiao Tong University (B2020013I).

### 2.2. Design and procedure

The experiment was conducted in a laboratory room in the hospital. The Spatial Working Memory and Attention Test on Paired Symbols (SWAPS, [16]), developed by our group and collaborators, was adopted to evaluate the performance of spatial WM. This test has been demonstrated as simple and appropriate for clinical use. 

**Figure 1:**
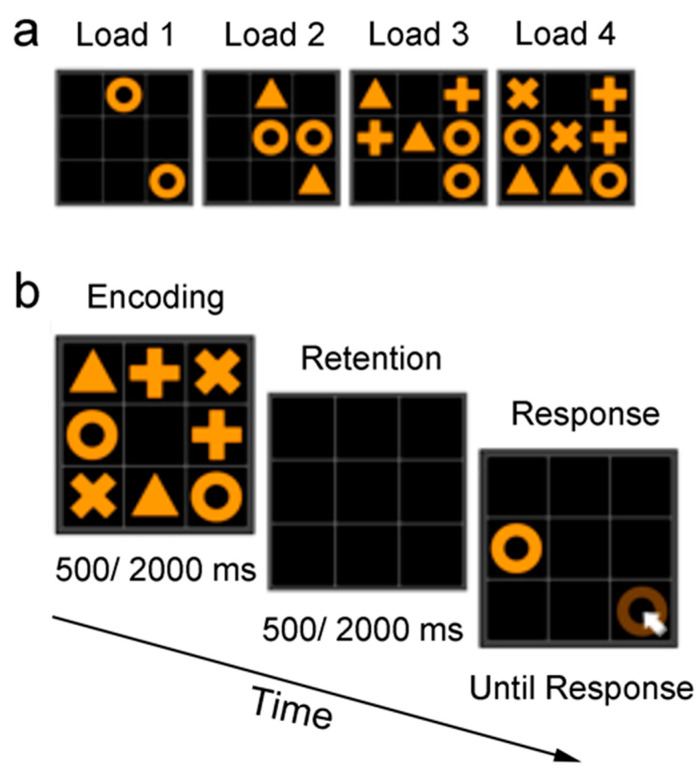
Stimuli (**a**) and the workflow of an example trial (**b**). Memory load was manipulated by varying the number of shape pairs. The circle and white arrow in the right–bottom cell is illustrated to show the correct response for the current trial but was not presented in the experiment.

The SWAPS test is composed of a visual two-dimensional grid plane (13° * 13° of visual angle) on a pad screen (Figure 1). At the beginning of each trial, two target shapes (each 3.4° * 3.4° of visual angle) located in two different cells in the grid were presented. Participants were asked to memorize the location of the presented items (i.e., the encoding phase). In different trials, the memory load was manipulated at different levels by varying the amount of the items. In the Load 1 condition, only two items with the same shape were presented. From Load 2 to Load 3 and Load 4, the number of the same-shape pairs increased from 2 to 3 and 4; there were two pairs of shapes in Load 2, three pairs of shapes in Load 3, and four pairs of shapes in Load 4. The time for encoding was either 500ms (short stimulus encoding) or 2000ms (long stimulus encoding). Then, an empty grid was presented, serving as the retention interval. The duration of the retention interval could be either 500ms (short interval) or 2000ms (long interval). After the retention interval, one of the memorized shapes was presented and participants were asked to indicate the correct location of the other item of the same shape by screen touching with the right index finger. The trial was not terminated unless a response was given. Participants were required to respond as accurately as possible. Load 1 and Load 2 were filler conditions, each of which included only 4 trials (The pilot study where Load 2 was included as an experimental condition showed the same pattern of results, see Supplementary Materials). For Load 3 and Load 4, there were 32 trials for each of conditions: stimuli-short (Encode-S), stimuli-long (Encode-L), interval-short (Interval-S), and interval-long (Interval -L). Trials under different conditions were mixed and presented in a random order. Prior to the formal experiment, an illustrated instruction was presented, and participants were required to complete five practice trials.

### 2.3. Statistical Analysis

For each participant, the accuracy (percentage of trials with correct response) and reaction times (RTs) in each experimental condition were calculated. The mean accuracy and RTs with standard error in each experimental condition is shown in Table 2. A 2 (group: MDD vs. Control) * 2 (load: Load 3 vs. Load 4) * 2 (encoding time: short vs. long) * 2 (retention interval: short vs. long) repeated-measures analysis of variance (ANOVA) was performed, with each group as the between-subjects factor. Further separate ANOVAs and *t* tests were conducted following an interaction with the groups. 

The accuracy and RT in each condition are shown in Table 2. Statistical inference mainly focused on accuracy because only correct responses, but not fast responses, were encouraged. However, to show if the pattern of RTs was consistent with accuracy, the same statistical analysis was also conducted on RTs.

We used α = 0.05 as the threshold for statistical significance. However, for two-way interactions that involved groups, further analyses were also conducted following a *p* value decrease of between 0.05 and 0.1. We chose to run further analyses following these effects based on our hypothesis that the performance in depression would be more affected by task difficulty (e.g., higher load, shorter encoding time, longer retention time). In cases of making a “no-difference” inference, a Bayes factor (BF) analysis was performed to quantify the extent to which the null hypothesis was more likely to be true than the alternative hypothesis [17,18]. Per convention, a BF > 3 is taken as moderate evidence for the tested hypothesis [19].

## 3. Results

**Figure 2:**
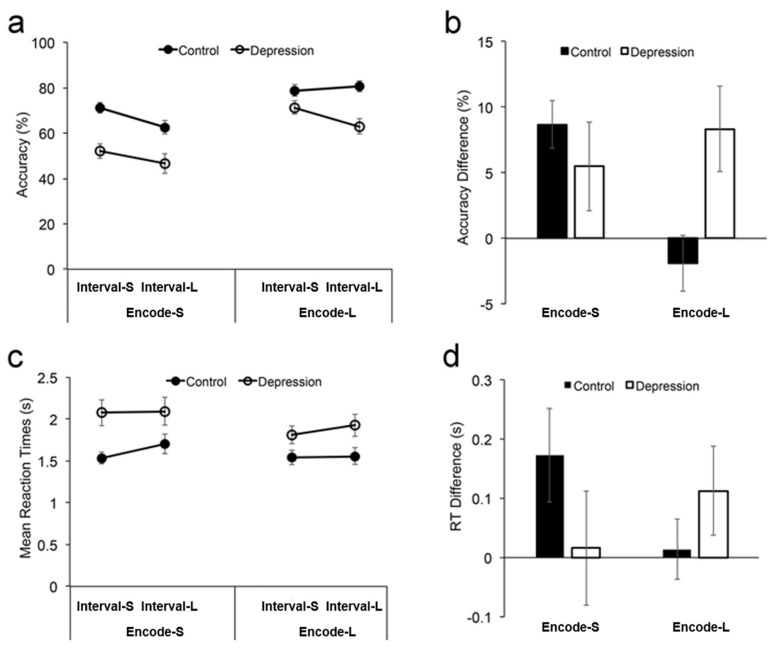
Accuracies (**a**) and mean reaction times (RTs) (**c**) with standard errors shown as a function of encoding time and retention time for each group. The difference in accuracy between short and long retention (**b**), and the difference in RT between long retention and short retention (**d**) with standard errors shown as a function of the encoding time for each group. Encode−S: short time for stimulus encoding; Encode−L: long time for stimulus encoding; Interval−S: short retention interval; Interval−L: long retention interval.

The four-way ANOVA on accuracies revealed the main effect of group, F(1, 67) = 17.60, *p* < 0.001, indicating a lower accuracy in the depression group (58.3%) compared with the control group (73.3%), *η_p_*^2^ = 0.208 (Figure 2a). The main effect of load was significant, F(1, 67) = 329.36, *p* < 0.001, indicating a lower accuracy under Load 4 (54.1%) than under Load 3 (77.4%), *η_p_*^2^ = 0.831. The main effect of encoding was significant, F(1, 67) = 113.66, *p* < 0.001, indicating a lower accuracy following a short encoding time (58.1%) than following a long encoding time (73.4%), *η_p_*^2^ = 0.629. The main effect of retention was also significant, F(1, 67) = 12.09, *p* < 0.001, indicating a lower accuracy after a long retention interval (63.2%) than after a short retention interval (68.4%), *η_p_*^2^ = 0.153. The interaction between group and load was significant, F(1, 67) = 6.75, *p* = 0.012, *η_p_*^2^ = 0.092. This interaction was due to the larger decreased accuracy by Load 4 (vs. Load 3) in the depression group (26.7%) compared with control group (20.0%), *t*(67) = 2.60, *p* = 0.012, Cohen’s d = 0.631, 95% confidence interval (CI) = (1.6%, 11.8%).There was also a significant interaction between load and encoding, F(1, 67) = 10.64, *p* = 0.002, *η_p_*^2^ = 0.137. However, the other two-way interactions did not reach significance (all p > 0.083). Moreover, the three-way interaction between groups, encoding, and retention was significant, F(1, 67) = 9.32, *p* = 0.003, *η_p_*^2^= 0.122, whereas the other three-way interactions did not reach significance, *p* > 0.192. The four-way interaction did not reach significance, F(1, 67) = 3.10, *p* = 0.083. 

Given the three-way interaction that involved the groups, encoding, and retention, a further analysis focused on teasing apart how retention was affected by groups and encoding, with the accuracy declining under Load 3 and Load 4. For this purpose, a separate 2 (group: MDD vs. control) * 2 (retention time: short vs. long) ANOVA was conducted for short and long encoding, respectively. Note that we did not examine how the encoding performance was affected by retention and group because encoding always precedes maintenance. 

For short encoding, both the main effect of group, F(1, 67) = 16.80, *p* < 0.001, *η_p_*^2^ = 0.200, and the main effect of retention, F(1, 67) = 15.37, *p* < 0.001, *η_p_*^2^ = 0.187, were significant, whereas the interaction between group and retention did not reach significance, F < 1, suggesting that the decreased accuracy by long retention (vs. short retention) was equivalent between the depression group (5.5%) and the control group (8.7%). For long encoding, while the main effect of retention was not significant, F(1, 67) = 2.93, *p* = 0.091, both the main effect of group, F(1, 67) = 13.80, *p* < 0.001, *η_p_*^2^ = 0.171, and the interaction between group and retention, F(1, 67) = 7.37, *p* = 0.008, *η_p_*^2^ = 0.099, were significant. This interaction occurred because only the depression group showed a significantly decreased accuracy by long retention (vs. short retention, 8.3%), paired *t*(29) = 2.56, p = 0.016, Cohen’s d = 0.467, 95%CI = (1.7%, 15.0%), whereas the control group showed no significant difference (–1.9%), *t* < 1. 

Moreover, the analysis of the accuracy difference showed that the decreased accuracy by long retention was larger following short encoding than long encoding in the control group (paired *t*(38) = 4.06, *p* < 0.001, Cohen’s d = 0.650, 95%CI = (5.3%, 15.8%)), whereas a decreased accuracy by long retention was equivalent between short encoding and long encoding in the depression group, *t* < 1 (Figure 2b). This lack of difference in the depression group was further confirmed by a BF analysis yielding B_01_ = 3.937, suggesting that the null hypothesis, i.e., “the decreased accuracies by long retention interval did not differ between short encoding and long encoding”, is 3.937 times more likely to be true than the alternative hypothesis, i.e., “the decreased accuracies by long retention interval was different between short encoding and long encoding”. In addition, although the accuracy of the depression group under short encoding and long retention was low, it was still above chance level (12.5%, one out of the other eight cells in the grid), *t*(29) = 10.70, *p* < 0.001 (one-sample *t* test), Cohen’s d = 1.96, 95%CI = (37.7%, 55.5%). These results suggest that the retention performance in the depression group was not further impaired by a short encoding time cannot be simply due to a floor effect.

The four-way ANOVA on RTs showed the main effect of group (Figure 2c), F(1, 67) = 6.62, *p* = 0.012, *η_p_*^2^ = 0.090, with slower responses in the depression group (1.98s) than responses in the control group (1.59s); the main effect of load, F(1, 67) = 22.93, *p* < 0.001, *η_p_*^2^ = 0.255, with slower responses under Load 4 (1.90s) than Load 3 (1.66s); the main effect of encoding, F(1, 67) = 14.06, *p* < 0.001, *η_p_*^2^ = 0.173, with slower responses following short encoding (1.85s) than following long encoding (1.71s); the main effect of retention, F(1, 67) = 4.20, *p* = 0.044, *η_p_*^2^ = 0.059, with slower responses after long retention (1.82s) than after short retention (1.74s). There was a trend of interaction between groups and encoding, F(1, 67) = 3.67, *p* = 0.060, *η_p_*^2^ = 0.052, which was due to a slower response by long encoding (vs. short encoding) in the depression group (218ms) than in the control group (71ms), *t*(67) = 1.92, *p* = 0.060, Cohen’s d = 0.465, 95% CI = (-6ms, 301ms). The interaction between load and retention was significant, F(1, 67) = 6.15, *p* = 0.016, *η_p_*^2^ = 0.084, whereas the other two-way interactions did not reach significance (all *p* > 0.308). There was a significant three-way interaction between load, encoding, and retention: F(1, 67) = 4.72, *p* = 0.033, *η_p_*^2^ = 0.066. No other significant effects were observed (all *p* > 0.091). Thus, the pattern of RTs was consistent with the pattern of accuracies in that the WM encoding was impaired in depression relative to the healthy controls. Although the RTs did not show a statistically significant interaction between groups, encoding, and retention, the pattern was the same as the pattern of accuracy (Figure 2d), ruling out the potential accuracy–speed trade-off in leading to the observed effects. The mean accuracies and reaction times in each experimental condition for each group are shown in Table 2.

## 4. Discussion

Similar to the WM deficits shown in previous studies on depression [9,12], the overall accuracy in the spatial WM task shown in this study was lower in the depression group compared with the healthy controls. In an extended study, we disentangled the components of the encoding and maintenance of WM and demonstrated the encoding and maintenance characteristics in recurrent MDD. Specifically, the depression group suffered more from the short encoding time than the control group, showing a larger decreased accuracy and more delayed response in the short encoding than the long encoding time. However, the difference between short and long retention in two groups was modulated differently by the encoding time. In the long encoding time, the depression group showed larger decreased accuracy by long retention (vs. short retention) than the control group. In the short encoding time, the decreased accuracy by long retention (vs. short retention) was increased in the control group, while the depression group was not further affected. Taken together, the results suggest that the encoding for WM was generally impaired in recurrent MDD relative to the healthy controls. By contrast, although the maintenance in depression was subject to long retention intervals, this maintenance was immune to the encoding constraints.

The encoding deficits in depression shown in this study align with the well-documented attentional deficits in depression [4]. As a system with limited capacity, WM relies on focal attention such that task-relevant information can be prioritized and task-irrelevant information can be effectively filtered out [20,21]. The demand for focal attention can be high when the encoding time is short (e.g., 500ms), resulting in a decreased performance relative to long encoding time (e.g., 2000ms), especially for the depression group whose focal attention is vulnerable. 

While the encoding in depression was generally impaired relative to the controls, the maintenance of the two groups showed different patterns following long encoding and short encoding. It has been suggested that the breadth of the current attentional focus was critically affected by mood [22,23]. For instance, attentional field was found to be narrowed by faces with negative emotion whereas broadened by faces with positive emotion [23]. Thus, depressed patients may have a narrowed attentional focus due to low mood. In agreement with this prediction, de Fockert and Cooper [24] found that participants with low depression scores showed a more efficient perceptual processing of global visual information than local information, whereas participants with high depression scores did not show this global bias, although they generally exhibited perceptual deficits. Based on these findings, in the present study, healthy controls may tend to encode all stimuli into WM. The resolution of the individual stimulus, however, could be lowered by the short encoding time. Importantly, the low resolution of the stimulus held in WM could further suffer from the long retention interval, resulting in more recall failures after long retention than after short retention. By contrast, the narrow attentional focus of the depression group may have only been allowed to be encoded a few times, which prevented the following retention further suffering from the short encoding time.

The WM maintenance implies that immunity to the encoding constraint could be related to enhanced internal processing, such as rumination, which is diagnostic for depression [25,26]. Joormann et al. (2011) [27] found that it was more difficult for depressive participants to change the order of items in WM, leading to a larger sorting cost than in healthy controls. Importantly, such sorting costs in depression were highly correlated with rumination score. Although their findings were specific to the items with negative emotion but not to the positive emotion, another study showed that the switch cost in depression occurred regardless of the emotional valence of the WM content [28]. This lack of flexibility in changing the WM content led to cognitive cost, which could prevent false memories. In this context, the maintenance immune-to-encoding constraint suggests that fixed internal processing in depression is not necessarily restricted to negative thoughts.

Attention and WM are closely related to each other [20,29]. As a cognitive control process, WM often shares overlapping neural circuits with top-down attention [30]. Postle et al. found that SWM (spatial working memory) and attention ability are supported by overlapping nerve bases, including the inferior parietal lobe, superior parietal lobe and lateral prefrontal lobe [31]. Although the encoding quality is mainly dependent on the attentional processing of the external stimuli, holding the encoded items during the retention period is suggested to be an internal attention process [29,32]. From this perspective, the WM encoding and maintenance characteristics in depression can be explained by the bias of internal attention over external attention and the inflexibility of coordinating between internal and external attention, which originate from the common, limited cognitive resource. Specifically, the lack of external attention led to a generally impaired encoding performance. When the encoding time was long and more external attention elements are generated, internal attention may have to be utilized to compensate for the lack of external attention. Then, the lack of flexibility in directing the cognitive resource back to support the internal maintenance led to impaired maintaining performance. By contrast, when the encoding time was short, there may be no time to direct attention from inside to outside, and the fixed internal attention prevents the maintenance from further suffering from the long retention intervals. 

The common resources of external and internal processing are also reflected in recent studies. Keller et al. suggested that patients with depression usually show selective attention impairment, sustained attention impairment, and divided attention impairment, which are based on the distribution of external attention. The maintenance of internal attention is usually manifested in the bias towards negative information [33]. Murphy et al. suggested stronger internal processing during retention but weaker external processing during encoding. The simultaneous impaired encoding could be counteracted by the enhanced maintenance in MDD, resulting in a comparable recall performance with the control group [34].

This study has a few limitations. Firstly, to evaluate the abnormal characteristics of basic encoding and maintenance processes of WM in depression, we used neutral stimuli but not emotional stimuli in the task. Therefore, this cannot explain the cognitive bias for negative information that was observed in the depressive population [4,5]. Secondly, the different levels of encoding and maintenance might have unequal power in distinguishing between the two groups [35]. The encoding and maintenance characteristics in recurrent MDD shown in this study should be further verified in tasks with matched discriminating power. Thirdly, we did not discuss and analyze the influence of drugs on cognitive performance, which can reveal the potential relationship between drugs and cognitive performance in the future.

## 5. Conclusions

By directly manipulating the difficulty of WM encoding and maintenance, we found that recurrent MDD showed encoding deficits that were more susceptible to limited encoding time compared to healthy controls. Importantly, while WM maintenance in healthy controls was easily affected by limited encoding time, the WM maintenance in recurrent MDD was immune to the constraints of encoding. The concurrently impaired MW encoding and limited maintenance that immunity to encoding constraints may reflect a common cognitive bias for fixed internal processing over external processing in depression. This common cognitive bias can serve as an integrated explanation for the abnormal WM encoding and maintenance processes in depression and is related to the rumination in depression. Our paradigm provided in this study can be a convenient and efficient test to investigate such cognitive processes during clinical diagnosis.

## Figures and Tables

**Table 1:** Demographic and clinical characteristics of participants (mean ± SD).

	Control	Depression	Statistics
Gender (F/M)	26/13	23/7	χ^2^ (1) = 0.82
Age (years)	30.4 ± 9.2	31.4 ± 13.1	*t*(67) = 0.38
BDI score	5.0 ± 4.1	24.0 ± 12.6	*t*(67) = 8.85, *p* < 0.001
Medication (yes/no)	0/39	30/0	
Total duration of medication (months)		22.4 ± 23.7	

BDI: Beck Depression Inventory.

**Table 2:** Mean accuracies (M) and reaction times (RT) with standard errors (SE) in each experimental condition for each group. Encode-S: short time for stimulus encoding; Encode-L: long time for stimulus encoding; Interval-S: short retention interval; Interval-L: long retention interval.

			Depression Group				Control Group			
			M (%)	SE (%)	RT (s)	SE (s)	M (%)	SE (%)	RT (s)	SE (s)
Load 3	Encode-S	Interval-S	69.4	4.0	1.90	0.16	82.6	2.7	1.37	0.06
		Interval-L	58.9	5.0	2.16	0.26	76.2	3.6	1.60	0.12
	Encode-L	Interval-S	81.9	3.4	1.64	0.09	85.8	2.6	1.40	0.08
		Interval-L	76.1	4.1	1.80	0.15	88.6	2.4	1.44	0.09
Load 4	Encode-S	Interval-S	34.8	3.9	2.25	0.18	59.9	3.0	1.70	0.09
		Interval-L	34.3	4.4	2.03	0.13	49.0	3.6	1.82	0.13
	Encode-L	Interval-S	60.7	3.3	1.98	0.14	71.7	3.2	1.69	0.11
		Interval-L	49.9	3.5	2.05	0.14	72.7	3.5	1.68	0.12

## Data Availability

The data presented in this study are available from the corresponding author.

## Supplementary Materials

The following supporting information can be downloaded at: https://www.mdpi.com/article/10.3390/brainsci13010038/s1, Figure S1: Data from the pilot study.
