# Supplementary material for: Decomposing Working Memory in Recurrent Major Depression: Impaired Encoding and Limited Maintenance Immune-to-Encoding Constraint
Source: Brain Sci. 2022 Dec 24;13(1):38. doi: 10.3390/brainsci13010038 (PMC9856303; doi:10.3390/brainsci13010038)
Supplement: Supplementary file 1 [file brainsci-13-00038-s001.zip › brainsci-1968268-supplementary.pdf]

Supplementary materials:

Results of Pilot Study

A total of 69 patients with recurrent MDD (41 females) and 114 healthy controls (54 females) participated in the pilot study. The experiment was the same as the main text with the following exceptions. Firstly, Load 2 was also included as an experimental condition in addition to Load 3 and Load 4, and there were 24 trials in each of the sub-conditions. Secondly, the stimuli were presented on a computer screen and participants were required to make their response by a mouse click. The results showed the same pattern as the results in the main text (see Supplementary Figure S1) regardless of Load 2 was included in (Figure S1a, S1b) or excluded from (Figure S1c, S1d) data analysis, suggesting the reliability of the results.

The required sample size for the formal experiment was estimated based on the group difference in accuracy in the pilot study. First, the independent *t* test comparing the accuracies (collapsed over Load, Encoding time and Retention time) between the two groups showed that $t(181) = 7.05$, $p < 0.001$, effect size in Cohen's d = 1.08. The achieved power calculated with G-power 3.1 [1] was 0.99, given $\alpha = 0.05$, effect size in d = 1.08, and sample size N1 = 69, N2 = 114. Based on the estimation with G-power 3.1, the required sample size was N = 33 for each group, given $\alpha = 0.05$, effect size in d = 1.08, power = 0.99.

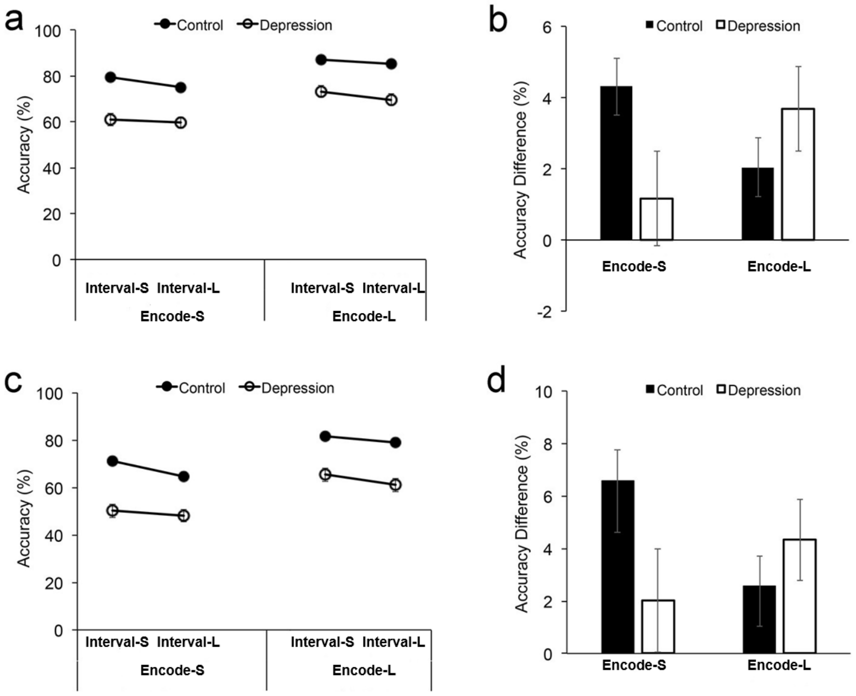

Figure S1. Data from the pilot study. (a, c) Accuracies with standard errors shown as a function of encoding time and retention time for each group. (b, d) The difference in accuracy between short retention and long retention with standard errors shown as a function of encoding time for each group. In (a, b), Load 2 was included, whereas in (c, d), Load 2 was excluded from the calculation. Encode−S: short time for stimulus encoding; Encode−L: long time for stimulus encoding; Interval−S: short retention interval; Interval−L: long retention interval.

Reference

1. Faul, F., E. Erdfelder, A.-G. Lang, and A. Buchner, G* Power 3: A flexible statistical power analysis program for the social, behavioral, and biomedical sciences. *Behav. Res. Methods*, 2007, 39, 175-191.
